# Supplementary material for: Comparative Safety of Bevacizumab, Ranibizumab, and Aflibercept for Treatment of Neovascular Age-Related Macular Degeneration (AMD): A Systematic Review and Network Meta-Analysis of Direct Comparative Studies
Source: J Clin Med. 2020 May 18;9(5):1522. doi: 10.3390/jcm9051522 (PMC7291235; doi:10.3390/jcm9051522)

**Supplementary Material 1. PRISMA NMA Checklist of Items to Include When Reporting A Systematic Review Involving a Network Meta-analysis.**

| Section/Topic | Item # | Checklist Item | Reported on Page # |
|---|---|---|---|
| **TITLE** | | | |
| Title | 1 | Identify the report as a systematic review *incorporating a network meta-analysis (or related form of meta-analysis).* | ***1*** |
| **ABSTRACT** | | | |
| Structured summary | 2 | Provide a structured summary including, as applicable: **Background:** main objectives. **Methods:** data sources; study eligibility criteria, participants and interventions; study appraisal and *synthesis methods, such as network meta-analysis.* **Results:** number of studies and participants identified; summary estimates with corresponding confidence/credible intervals; *treatment rankings may also be discussed. Authors may choose to summarize pairwise comparisons against a chosen treatment included in their analyses for brevity.* **Discussion/Conclusions:** limitations; conclusions and implications of findings. **Other:** primary source of funding; systematic review registration number with registry name. | ***2-3*** |
| **INTRODUCTION** | | | |
| Rationale | 3 | Describe the rationale for the review in the context of what is already known*, including mention of why a network meta-analysis has been conducted.* | ***3-5*** |
| Objectives | 4 | Provide an explicit statement of questions being addressed, with reference to participants, interventions, comparisons, outcomes and study design (PICOS). | 5 |
| **METHODS** | | | |
| Protocol and registration | 5 | Indicate whether a review protocol exists and if and where it can be accessed (e.g., web address), and, if available, provide registration information, including registration number. | 5 |
| Eligibility criteria | 6 | Specify study characteristics (e.g., PICOS and length of follow-up) and report characteristics (e.g., years considered, language and publication status) used as criteria for eligibility, giving rationale. *Clearly describe eligible treatments included in the treatment network and note whether any have been clustered or merged into the same node (with justification).* | ***5*** |
| Information sources | 7 | Describe all information sources (e.g., databases with dates of coverage and contact with study authors to identify additional studies) in the search and date last searched. | 5 |
| Search | 8 | Present full electronic search strategy for at least one database, including any limits used, such that it could be repeated. | 5 |
| Study selection | 9 | State the process for selecting studies (i.e., screening, eligibility, included in systematic review and, if applicable, included in the meta-analysis). | 6 |
| Data collection process | 10 | Describe method of data extraction from reports (e.g., piloted forms, independently, in duplicate) and any processes for obtaining and confirming data from investigators. | 7 |
| Data items | 11 | List and define all variables for which data were sought (e.g., PICOS and funding sources) and any assumptions and simplifications made. | 7 |
| **Geometry of the network** | **S1** | Describe the methods used to explore the geometry of the treatment network under study and potential biases related to it. This should include how the evidence base has been graphically summarized for presentation and what characteristics were compiled and used to describe the evidence base to readers. | ***7-8*** |
| Risk of bias within individual studies | 12 | Describe methods used for assessing the risk of bias of individual studies (including specification of whether this was done at the study or outcome level) and how this information is to be used in any data synthesis. | 7-8 |
| Summary measures | 13 | State the principal summary measures (e.g., risk ratio and difference in means). *Additionally, describe the use of additional summary measures* | 8 |

| | | | |
|---|---|---|---|
| | | *assessed, such as treatment rankings and surface under the cumulative ranking curve (SUCRA) values, as well as modified approaches used to present summary findings from meta-analyses.* | |
| Planned methods of analysis | 14 | Describe the methods of handling data and combining results of studies for each network meta-analysis. This should include but not be limited to:<br>• *Handling of multi-arm trials,*<br>• *Selection of variance structure,*<br>• *Selection of prior distributions in Bayesian analyses and*<br>• *Assessment of model fit.* | 8 |
| **Assessment of Inconsistency** | **S2** | Describe the statistical methods used to evaluate the agreement of direct and indirect evidence in the treatment network(s) studied. Describe efforts taken to address their presences when found. | 9 |
| Risk of bias across studies | 15 | Specify any assessment of the risk of bias that may affect the cumulative evidence (e.g., publication bias and selective reporting within studies). | **9** |
| Additional analyses | 16 | Describe methods of additional analyses if done, indicating which were prespecified. This may include, but not be limited to, the following:<br>• Sensitivity or subgroup analyses,<br>• Meta-regression analyses,<br>• *Alternative formulations of the treatment network and*<br>• *Use of alternative prior distributions for Bayesian analyses (if applicable).* | |
| **RESULTS†** | | | |
| Study selection | 17 | Give the number of studies screened, assessed for eligibility and included in the review, with reasons for exclusions at each stage, ideally with a flow diagram. | 8 |
| **Presentation of network structure** | **S3** | Provide a network graph of the included studies to enable visualization of the geometry of the treatment network. | |
| **Summary of network geometry** | **S4** | Provide a brief overview of characteristics of the treatment network. This may include commentary on the abundance of trials and randomized patients for the different interventions and pairwise comparisons in the network, gaps of evidence in the treatment network and potential biases reflected by the network structure. | ***9*** |
| Study characteristics | 18 | For each study, present characteristics for which data were extracted (e.g., study size, PICOS and follow-up period) and provide the citations. | 9 |
| Risk of bias within studies | 19 | Present data on the risk of bias of each study and, if available, any outcome level assessment. | 9 |
| Results of individual studies | 20 | For all outcomes considered (benefits or harms), present, for each study: (1) simple summary data for each intervention group and (2) effect estimates and confidence intervals. *Modified approaches may be needed to deal with information from larger networks.* | ***10*** |
| Synthesis of results | 21 | Present results of each meta-analysis done, including confidence/credible intervals. *In larger networks, authors may focus on comparisons vs. a particular comparator (e.g., placebo or standard care), with full findings presented in an appendix. League tables and forest plots may be considered to summarize pairwise comparisons.* If additional summary measures were explored (such as treatment rankings), these should also be presented. | ***10-11*** |
| **Exploration for inconsistency** | **S5** | Describe results from investigations of inconsistency. This may include such information as measures of model fit to compare consistency and inconsistency models, *p*-values from statistical tests or summaries of inconsistency estimates from different parts of the treatment network. | |
| Risk of bias across studies | 22 | Present results of any assessment of the risk of bias across studies for the evidence base being studied. | 9 |
| Results of additional analyses | 23 | Give results of additional analyses, if done (e.g., sensitivity or subgroup analyses, meta-regression analyses*, alternative network geometries studied, alternative choices of prior distributions for Bayesian analyses* and so forth). | |
| **DISCUSSION** | | | |
| Summary of evidence | 24 | Summarize the main findings, including the strength of evidence for each main outcome; consider their relevance to key groups (e.g., healthcare providers, users and policy-makers). | 11-15 |
| Limitations | 25 | Discuss limitations at study and outcome levels (e.g., risk of bias) and at the review level (e.g., incomplete retrieval of identified research and reporting bias). *Comment on the validity of the assumptions, such as* | 15 |

| | | | |
|---|---|---|---|
| | | *transitivity and consistency. Comment on any concerns regarding network geometry (e.g., avoidance of certain comparisons).* | |
| Conclusions | 26 | Provide a general interpretation of the results in the context of other evidence and implications for future research. | 17 |
| **FUNDING** | | | |
| Funding | 27 | Describe sources of funding for the systematic review and other support (e.g., supply of data) and role of funders for the systematic review. This should also include information regarding whether funding has been received from the manufacturers of treatments in the network and/or whether some of the authors are content experts with professional conflicts of interest that could affect the use of treatments in the network. | ***17*** |

PICOS = population, intervention, comparators, outcomes and study design. * Text in italics indicates wording specific to the reporting of network meta-analyses that have been added to the guidance from the PRISMA statement. † Authors may wish to plan for the use of appendices to present all relevant information in full detail for items in this section.

**Supplementary Materials 2. Risk of bias assessment.**

*Randomization and allocation*

VIEW 1,2 predetermined central randomization scheme with balanced allocation, managed by an interactive voice response system. CATT1 described the permuted-block method with a randomly chosen block size randomization; however, re-randomization after the one year of treatment is not described in detail. IVAN-reported computer-generated allocation concealed with an internet-based system that was used by responsible authorized staff accessing the website being provided with the unique study number. In Gefal, randomization was organized with subject allocation in each center using pre-established lists stratified by the center. In the BRAMD study, a randomization list was created by a specified computer system, the allocation scheme was stratified and a randomization list was then imported into the data management system. In the LUCAS study, a randomization process was computer-generated by a third party with the use of the block method and stratified by the center. In the MANTA study, a randomization was stratified according to the clinical center using a permuted-block method with a fixed block size by staff members of the Clinical Pharmacology Department not involved in the study otherwise. Biswas study used random number tables where 60 numbers were randomly picked up from 1 to 120 and assigned to groups A and B. Upon initiation of enrollment, the patients were numbered sequentially based on the serial order of enrolment in the study with automatic allocation to the treatment arm.

*Masking*

VIEW 1,2 maskings were performed using separation study records and drug packaging by unmasked personnel who managed study drug logistics, preparations and administration of the study drug and masked personal who performed assessment. However, the risk of patient unblinding still existed, since unmasked personal injected drugs using only masking packages. In CATT2, insurance and billing documents specified ranibizumab, so patients may have learned their assigned drug from these financial documents. In IVAN, generally, there was separation of unmasked pharmacy personnel who dispensed the study drug but had no other role in the study and several centers where unmasked nurses prepared syringes to get the same final appearance of the drug to be injected, which might have led to a risk of unmasking; however, a study survey showed that 98%-99% of staff/patients reported not knowing what was their study drug. In Gefal, identical syringes were masked and delivered by local hospital pharmacies after aseptic preparation in authorized, centralized drug-preparation units. In the BRAMD study, the data management system sent upon randomization automatized email notifications to the site's pharmacy, keeping the investigator and trial personnel blinded from treatment allocation. At the same time, in the Gefal and BRAMD studies, no specific data were given regarding the masking of staff involved in the outcome assessment. In LUCAS, the drugs were allocated by unmasked study nurses (not involved in any

other patient-related activity) responsible for the aseptic filling of identical syringes with the assigned drug behind a screen and presenting it to the treating ophthalmologist, so the patient, the treating ophthalmologist, assisting nurse and ophthalmic nurses testing the ETDRS visual acuity were masked. In the MANTA study, the drugs were prepared by the local pharmacies with an unblinded injecting physician not involved in the collection of data and an evaluating physician who was masked to the treatment assignment. The Biswas study did not provide any details on how patients, injecting physicians and assessors were masked, since it is unclear who handled the randomization tables and who prepared the syringes and how it was masked and whether the assessors were separate staff.

*Incomplete outcome data*

Incomplete safety reporting surprisingly seems to be the most unclear zone in terms of bias assessment. In the CATT2 study, ocular SAE were not published in detail. IVAN study had no data on endophthalmitis and pseudo-endophthalmitis, even in the appendix, and it is unclear whether there were no endophthalmitis in this study or it was recognized as another SAE, e.g., severe uveitis. The Gefal report on ocular SAE happened to be not full and, therefore, not fully clear. Most risk of bias in terms of reporting was revealed in the BRAMD study, since no details on SAE categories and causes of death were reported. We did not find data also on the total number of ocular SAE in the LUCAS studies. VIEW 1,2 had not fully detailed information on ocular SAE (pseudo-endophthalmitis and geographical atrophy); omitting ocular SAE appeared once in a study arm. The BRAMD study published only the total death, SAE and AE numbers per group without specifying information regarding splitting the systemic and ocular SAE, its categories and cause of death. In the MANTA study, the cause of death and details on dropout reasons were not reported.

*Selective reporting*

We have not found any sign of potential selective reporting in terms of safety endpoints of our interest; however, some discrepancies were identified between protocols and publications reporting before in the Gefal and IVAN studies; no Biswas protocol was available for this comparison [15].

*Other potential sources of bias*

VIEW 1,2 and Biswas surprisingly had no sample size calculations described clearly in their primary publications; however, VIEW study authors mentioned that the study was not powered enough to differentiate rare ocular SAE, whereas the Biswas reported that their sample size calculation was based on the literature data analysis BRAMD.

Summarized data on the potential risks of bias are presented below in Table S1.

**Table S1.** Potential risk of bias assessment of included RCTs. (Note: RIVAL 2-years study was not included into the risk of bias analysis, since the full manuscript was not published yet, only unpublished data generously provided by the study sponsor).

| | Random sequence generation (selection bias) | Allocation concealment (selection bias) | Blinding of participants and personnel (performance bias) | Blinding of outcome_assessment (detection bias) | Incomplete outcome data (attrition data) | Selective reporting (reporting bias) | Other bias |
|---|---|---|---|---|---|---|---|
| **CATT2** | + | ? | ? | + | ? | + | + |
| **VIEW 2** | + | + | ? | + | + | + | ? |
| **IVAN 2** | + | + | + | + | ? | + | + |
| **GEFAL** | + | + | + | ? | + | + | + |
| **BRAMD** | + | + | + | ? | - | + | + |
| **LUCAS 2** | + | ? | + | + | ? | + | + |
| **MANTA** | + | + | + | + | ? | + | + |
| **Biswas** | + | + | ? | ? | ? | + | ? |

**Supplementary Materials 3.** **Pairwise ranibizumab vs. bevacizumab adverse events incidence rate ratio.**

## Death rate

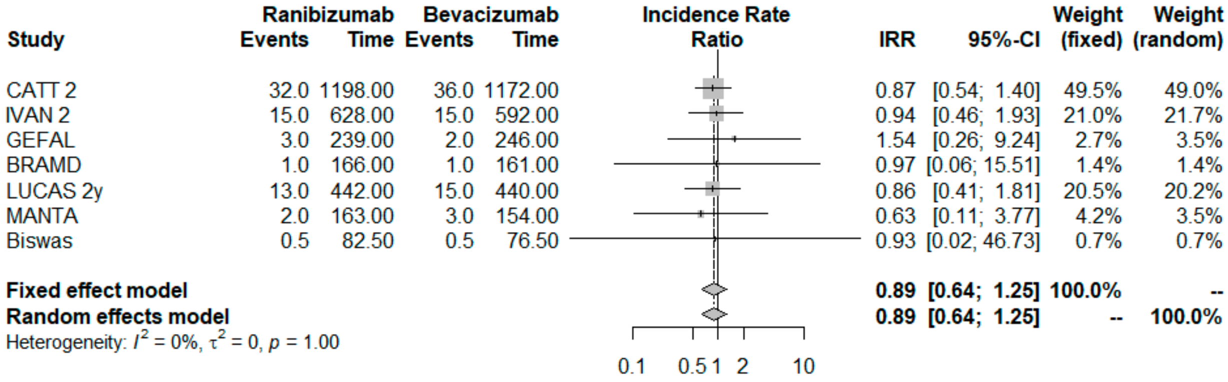


## Systemic SAE rate

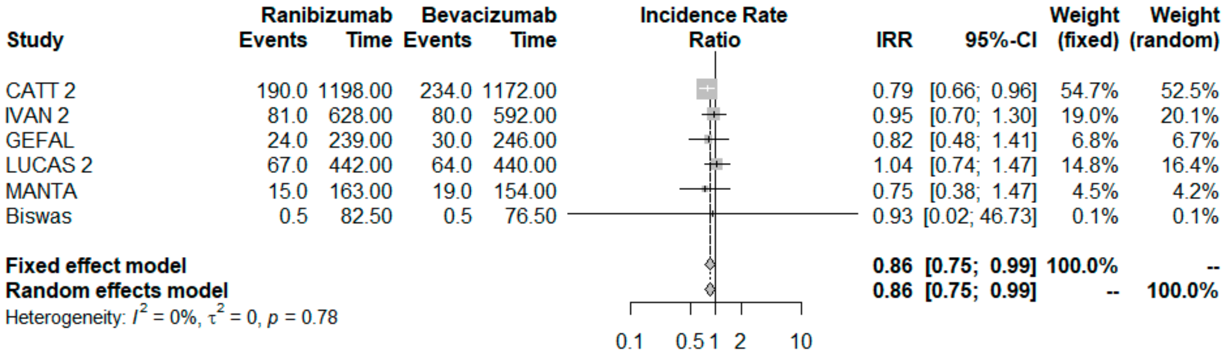


## CVD death rate

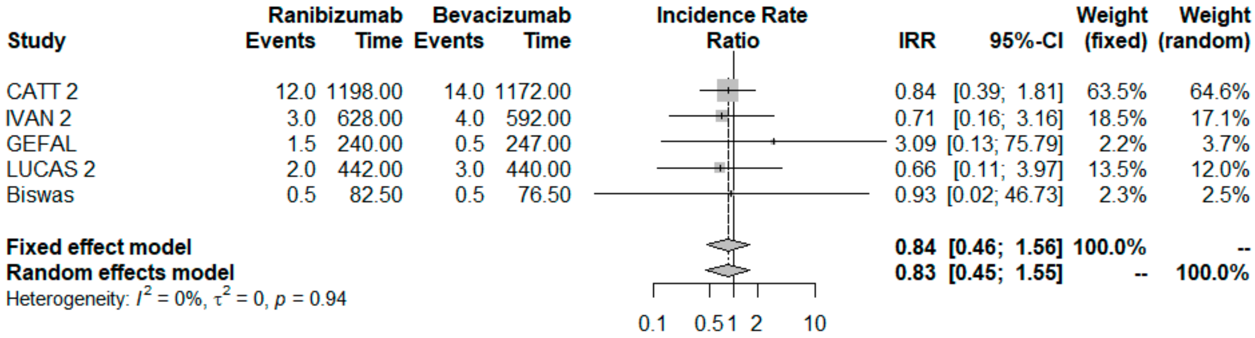


## Venous thrombotic events

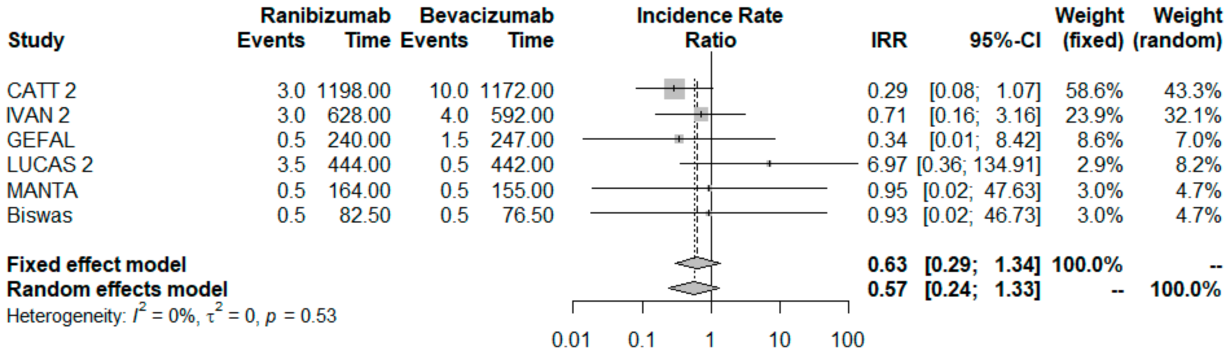


## Atherothrombotic events

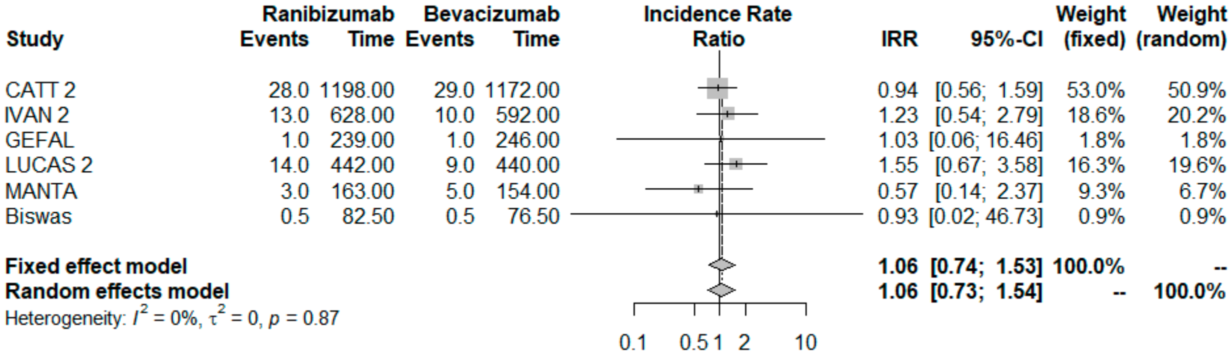


## Ocular SAE

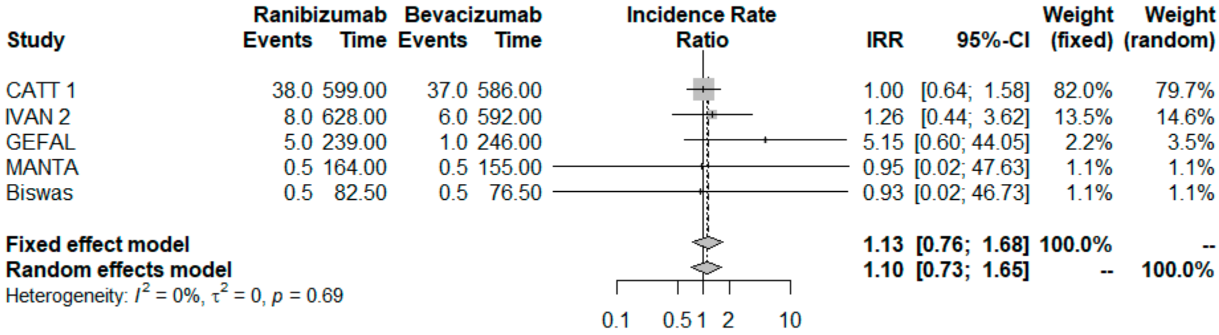


## Endophthalmitis

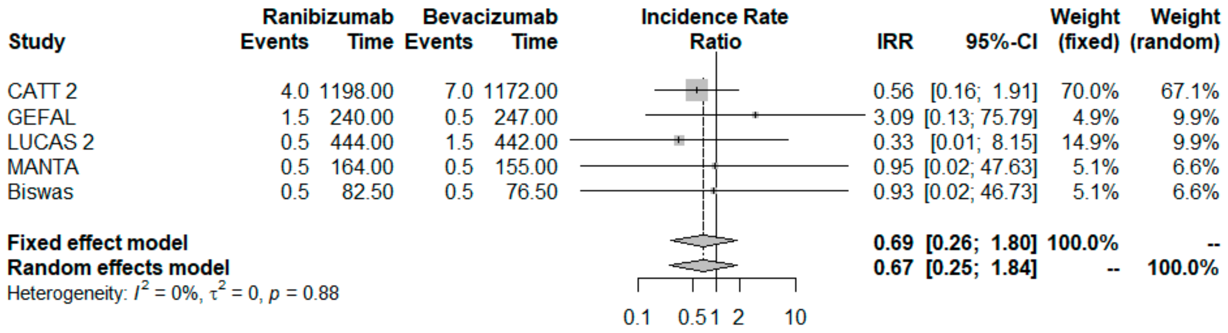


Pseudo-endophthalmitis

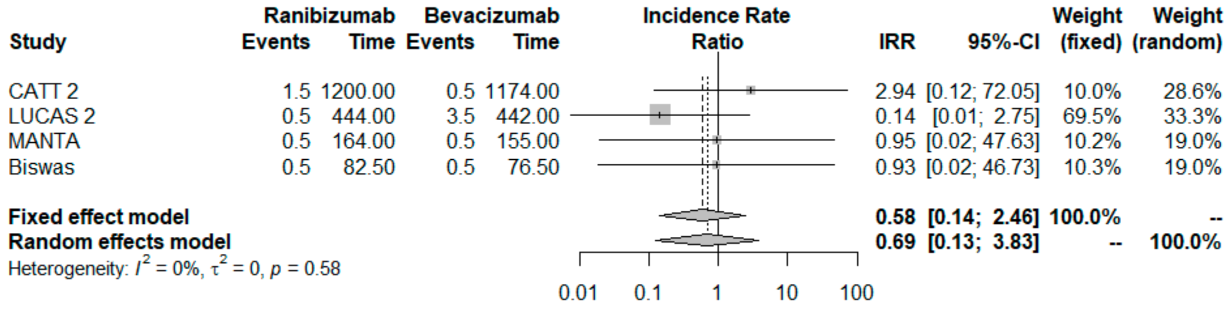


Retinal pigment tear

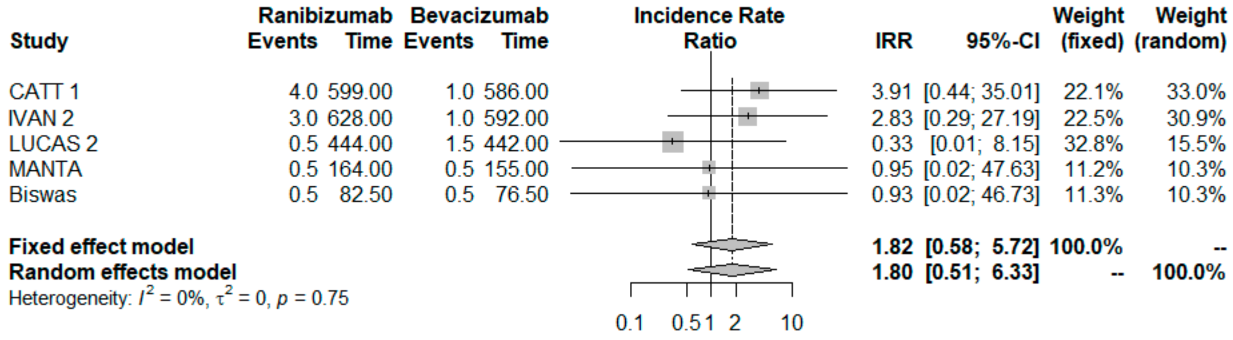


Newly identified macular (geographical) atrophy

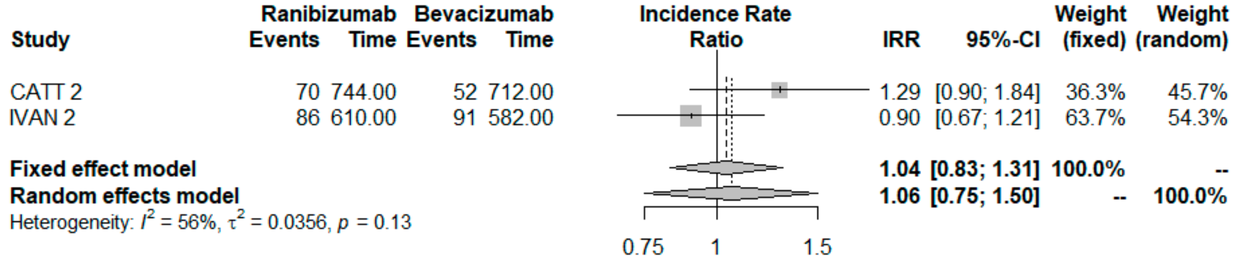


Overall dropout

| Study | Ranibizumab Events | Ranibizumab Time | Bevacizumab Events | Bevacizumab Time | IRR | 95%-CI | Weight (fixed) | Weight (random) |
|---|---|---|---|---|---|---|---|---|
| CATT 2 | 71 | 1198.00 | 84 | 1172.00 | 0.83 | [0.60; 1.13] | 27.0% | 25.7% |
| IVAN 2 | 43 | 628.00 | 42 | 592.00 | 0.97 | [0.63; 1.48] | 13.8% | 14.2% |
| GEFAL | 56 | 239.00 | 55 | 246.00 | 1.05 | [0.72; 1.52] | 17.3% | 18.6% |
| BRAMD | 29 | 166.00 | 34 | 161.00 | 0.83 | [0.50; 1.36] | 11.0% | 10.5% |
| LUCAS 2y | 49 | 442.00 | 53 | 440.00 | 0.92 | [0.62; 1.36] | 16.9% | 17.0% |
| MANTA | 36 | 163.00 | 33 | 154.00 | 1.03 | [0.64; 1.65] | 10.8% | 11.5% |
| Biswas | 6 | 90.00 | 10 | 90.00 | 0.60 | [0.22; 1.65] | 3.2% | 2.5% |
| **Fixed effect model** | | | | | **0.91** | **[0.78; 1.07]** | **100.0%** | **--** |
| **Random effects model** | | | | | **0.92** | **[0.78; 1.07]** | **--** | **100.0%** |

Heterogeneity: $I^2 = 0\%$, $\tau^2 = 0$, $p = 0.92$

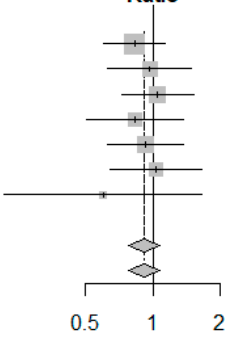


**Supplementary Materials 4.** **Pairwise ranibizumab vs. aflibercept adverse events incidence rate ratio (VIEW1,2 and unpublished RIVAL data).**

## Death rate

| Study | Ranibizumab Events | Ranibizumab Time | Aflibercept Events | Aflibercept Time | IRR | 95%-CI | Weight (fixed) | Weight (random) |
|---|---|---|---|---|---|---|---|---|
| VIEW1,2 | 16 | 1190.00 | 20 | 1220.00 | 0.82 | [0.43; 1.58] | 76.6% | 81.6% |
| RIVAL | 3 | 282.00 | 6 | 278.00 | 0.49 | [0.12; 1.97] | 23.4% | 18.4% |
| **Fixed effect model** | | | | | **0.74** | **[0.41; 1.34]** | **100.0%** | **--** |
| **Random effects model** | | | | | **0.75** | **[0.41; 1.35]** | **--** | **100.0%** |

Heterogeneity: $I^2 = 0\%$, $\tau^2 = 0$, $p = 0.52$

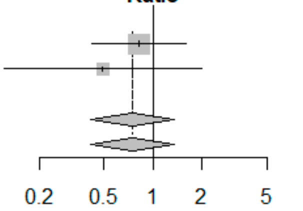


## Systemic SAE rate

| Study | Ranibizumab Events | Ranibizumab Time | Aflibercept Events | Aflibercept Time | IRR | 95%-CI | Weight (fixed) | Weight (random) |
|---|---|---|---|---|---|---|---|---|
| VIEW1,2 | 146 | 1190.00 | 154 | 1220.00 | 0.97 | [0.78; 1.22] | 73.7% | 74.3% |
| RIVAL | 50 | 282.00 | 54 | 278.00 | 0.91 | [0.62; 1.34] | 26.3% | 25.7% |
| **Fixed effect model** | | | | | **0.96** | **[0.79; 1.16]** | **100.0%** | **--** |
| **Random effects model** | | | | | **0.96** | **[0.79; 1.16]** | **--** | **100.0%** |

Heterogeneity: $I^2 = 0\%$, $\tau^2 = 0$, $p = 0.78$

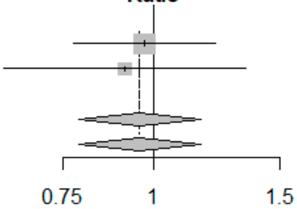


## CVD death rate

| Study | Ranibizumab Events | Ranibizumab Time | Aflibercept Events | Aflibercept Time | IRR | 95%-CI | Weight (fixed) | Weight (random) |
|---|---|---|---|---|---|---|---|---|
| VIEW1,2 | 3.0 | 1190.00 | 11.0 | 1220.00 | 0.28 | [0.08; 1.00] | 95.6% | 90.4% |
| RIVAL | 0.5 | 284.00 | 0.5 | 280.00 | 0.99 | [0.02; 49.69] | 4.4% | 9.6% |
| **Fixed effect model** | | | | | **0.31** | **[0.09; 1.03]** | **100.0%** | **--** |
| **Random effects model** | | | | | **0.32** | **[0.09; 1.06]** | **--** | **100.0%** |

Heterogeneity: $I^2 = 0\%$, $\tau^2 = 0$, $p = 0.55$

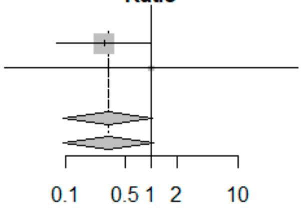

## Atherothrombotic events

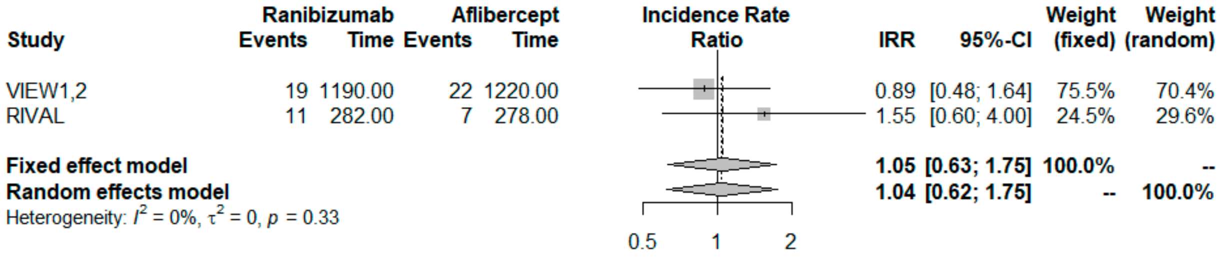


## Ocular SAE

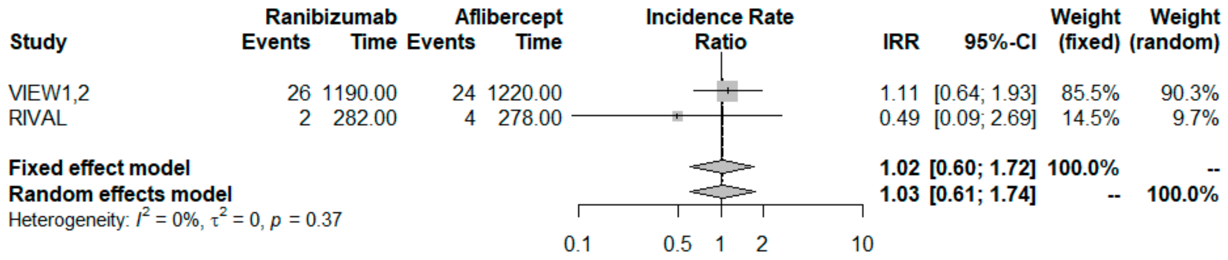


## Endophthalmitis

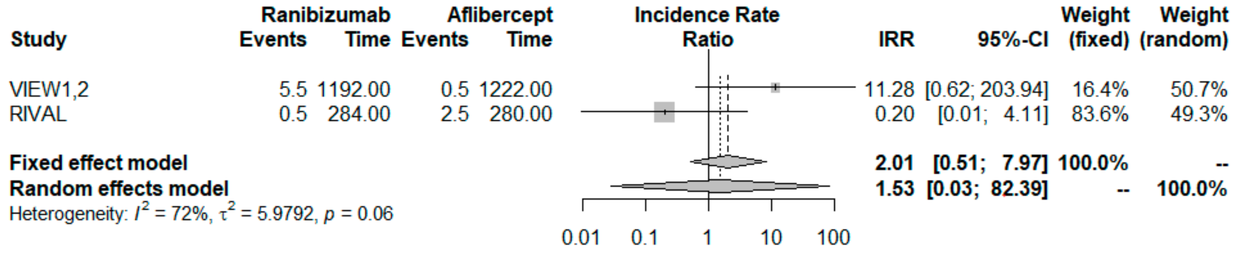


## Newly identified macular (geographical) atrophy

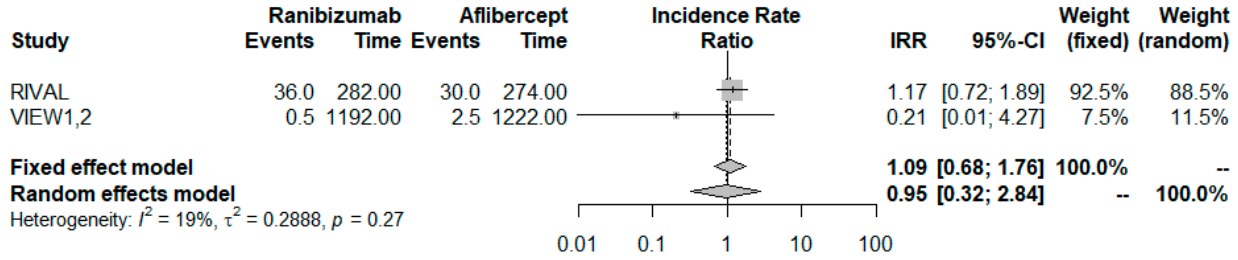


## Overall dropout

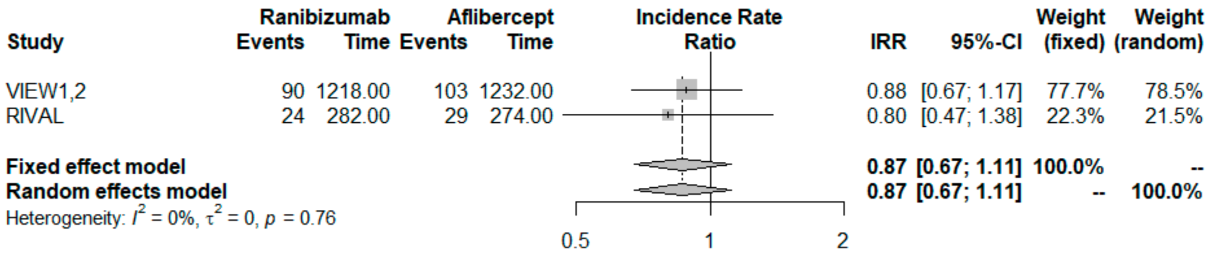


**Funnel plot of newly identified macular (geographical) atrophy in VIEW1,2 and RIVAL studies ($I^2$=19%).**

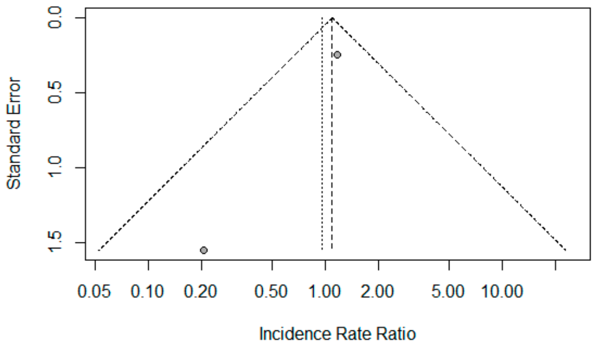


**Supplementary Materials 5.** **Network meta-analysis of adverse events related to aflibercept and bevacizumab compared to the ranibizumab reference.**

### Death rate

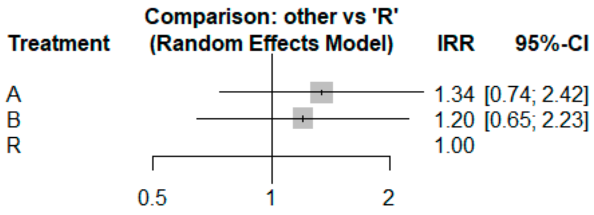


### CVD death rate

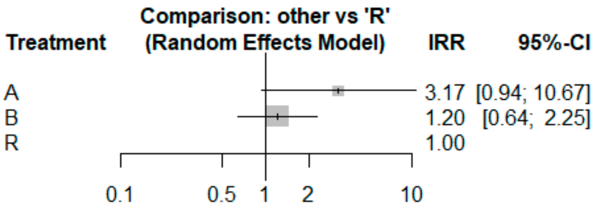


### Systemic SAE

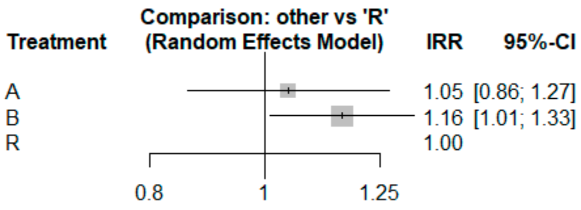


### Ocular SAE

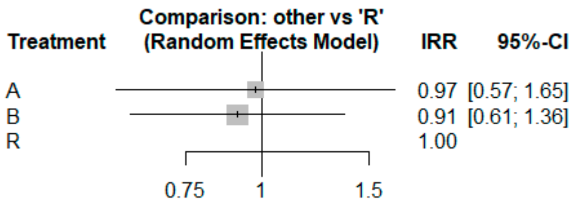


### Atherotrombotic events

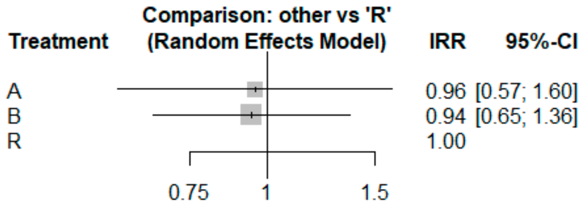


### Endophthalmitis

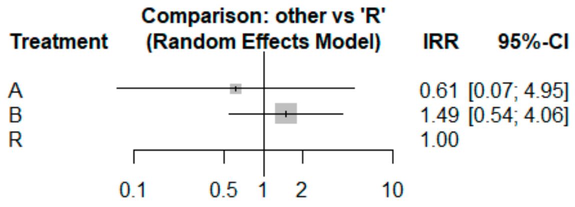

**Retinal tear**

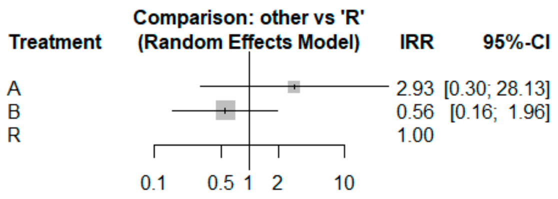


**Dropout**

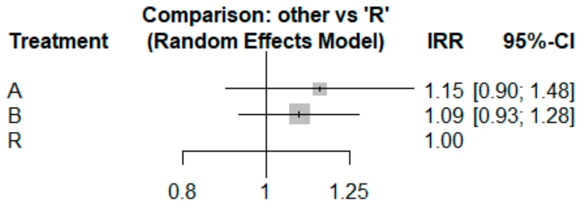

Supplement: Supplementary file 1 [file jcm-09-01522-s001.pdf]
